# King-Denborough Syndrome-Dysmorphic Features Suggesting Risk of Malignant Hyperthermia

**DOI:** 10.34763/jmotherandchild.20263001.d-26-00012

**Published:** 2026-06-28

**Authors:** Piotr Nowakowski, Karolina Czeczko, Anna Potulska-Chromik, Justyna Żelewska, Aleksandra Jastrzębska, Anna Wójcicka, Paweł Gaj, Anna Kostera-Pruszczyk

**Affiliations:** Student Scientific Circle, Department of Neurology, Medical University of Warsaw, Banacha 1a, 02-097 Warsaw, Poland; Department of Neurology, Medical University of Warsaw, Banacha 1a, 02-097 Warsaw, Poland, European Reference Network EURO-NMD; Warsaw Genomics Kiwerska 33A 01-682 Warsaw, Poland

**Keywords:** Rhabdomyolysis, muscle MRI, RYR1 mutations

## Abstract

**Background:**

King-Denborough syndrome is a rare disorder within the spectrum of RYR1-related myopathies. It is a congenital myopathy, with characteristic dysmorphic features, and susceptibility to malignant hyperthermia. Early recognition is clinically important because the diagnosis may affect anesthetic management and perioperative safety.

**Case presentation:**

We report a 4.5-year-old boy with delayed motor development, generalized hypotonia, characteristic dysmorphic features, and mildly elevated serum creatine kinase levels. The MRI showed features of asymmetrical atrophy and fatty degeneration in the proximal muscles of the lower limbs.

**Results:**

Molecular testing identified a heterozygous RYR1 variant. In the context of the clinical phenotype and muscle imaging findings, the presentation was considered suggestive of King-Denborough syndrome within the spectrum of RYR1-related disorders.

**Conclusion:**

This case highlights the importance of another RYR1-related myopathy in children presenting with myopathic features, dysmorphism, and muscle MRI abnormalities. Recognition of this phenotype is particularly important because of the potential risk of malignant hyperthermia and the need for appropriate anesthetic precautions.

## Introduction

King Denborough syndrome (KDS, OMIM 619542) is a rare, congenital myopathy associated with susceptibility to malignant hyperthermia, skeletal abnormalities and dysmorphic features with characteristic facial features. This syndrome was first described by King and Denborough in 1973 (1), while analyzing 19 cases of malignant hyperthermia, they noticed four male patients with very similar appearance. All the patients presented short stature, lumbar lordosis, thoracic kyphosis, pectus carinatum, cryptorchidism. Moreover, they shared one or more facial features, such as hypoplastic mandible, low attachment of ears, obliquity of palpebral fissures, ptosis or webbing neck. Over the following years, further similar cases were reported (2,3,4), in addition to malignant hyperthermia (MH) susceptibility and dysmorphia, in some patients myopathic features were described (4, 5). Concluding that KDS is a type of myopathy, it was supported by various myopathic changes in muscle biopsies (4). The disease is predominantly associated with various mutations in *RYR1* gene encoding the skeletal muscle ryanodine receptor. It plays a relevant role in excitation-contraction coupling, and its dysfunction is associated with several neuromuscular diseases (6). To date, there have been reports suggesting both the recessive and dominant inheritance manner (7, 8), therefore a genetic basis of KDS is not fully understood (8).

We present a case of a patient with suggestive symptoms of KDS.

## Case report

The boy was born at 38 Hbd by a cesarean section, presenting with hypotonia, facial dysmorphic features and cryptorchidism and delayed motor development. He started independent walking at 18-months, with incorrect posture and hyperlordosis. The Noonan and the Silver-Russel syndromes, were excluded.

At the age of 4.5, the patient was first seen at our Department of Neurology.

He presented with deficiency in growth and weight, bilateral ptosis, clinodactyly of the fifth finger of both hands and severe hyperlordosis. Neurological examination showed generalized hypotonia, without obvious deficits of muscle strength. There was no family history of neurological diseases or adverse reaction to general anesthesia. Laboratory investigations showed elevated CK (max. 422 U/l, range 39-308U/l). Electromyography was normal. KDS was suspected due to characteristic appearance.

Molecular analysis was performed on genomic DNA isolated from peripheral blood according to Miller at al. Targeted NGS sequencing was performed for the panel of 127 genes associated with dystrophies and elevated CK level (list Supp. 1). Analysis covered the coding exons of the selected genes and the flanking 10–20 intronic nucleotides. Enrichment and sequencing were performed with standard Illumina protocols using the NextSeq500 platform (read length: 2 × 75 bp). The mean sequencing coverage depth was 93.98% at the quality threshold 98.3%. Variants were identified using the Burrows–Wheeler Aligner with reference to the human genome (GRCh37).

NGS data analysis revealed presence of the heterozygous substitution in the *RYR1* gene - the substitution c.11933G>A (NM_001042723) resulting in a missense variant p.Arg3978His [according to current GRCh38 canonical MANE Selectref. sequence c.11948G>A, p.Arg3983His; patients’genotype NM_000540.3, c.[11948G>A];[11948=], NP_ 000531.2; p.[Arg3983His];[Arg3983=].

Identified variant was rare – gnomAD v.3.1.2 = 0, with no match in dbSNP v157 and ClinVar v20260329, in HGMD Professional 2026.1 reported in context of MH as VUS (CM1010055). Aggregated prediction of pathogenicity based on CADD phred (24.50) and REVEL (deleterious, 0.93) classified the variant as pathogenic.

Identified variant in the *RYR1* gene was classified according to ACMG Guidelines as Likely Pathogenic [PM2, PP3, PP2, PM6].

Targeted sequencing (NGS) of the *RYR1* gene for the proband and his parents revealed *de novo* origin of the variant. Analysis performed for the proband’s healthy brother (parental germline mosaicism is possible), excluded its presence. IGV of the mutation is presented in Figure 1. The whole-body MRI (performed on 1.5 T Siemens Avanto scanner, Figures 2-6) showed complete fatty atrophy in gluteus maximus muscles, asymmetric muscle atrophy at the thigh level in hamstrings, with relative sparing of the right semitendinosus muscle just below ischial tuberosity. Relative hypertrophy of the short head of biceps femoris muscle was observed bilaterally. Patient presented with intact anterior muscle compartment at the thigh level with relative hypertrophy of the sartorius and the gracilis muscles. In the lower legs muscles showed no fatty infiltration with relative hypertrophy of the soleus and the gastrocnemius muscles. Paraspinal muscles and upper limbs muscles were spared. CK was still slightly elevated.

**Figure 1.**
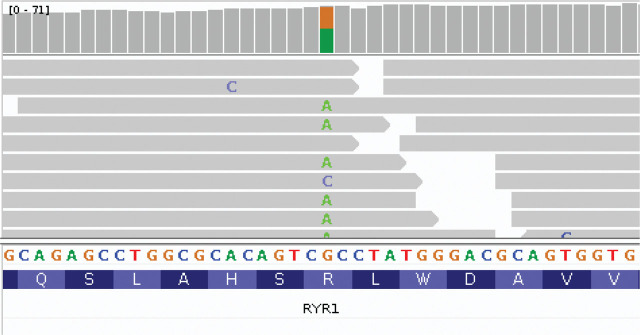
IGV of the mutation.

**Figure 2.**
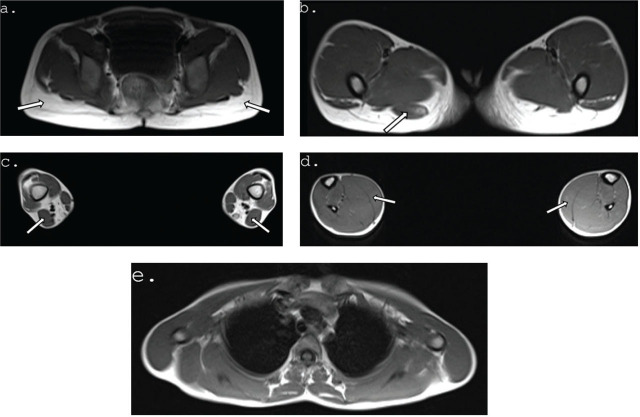
Magnetic Resonance Imaging (MRI). **(A-E)** Selected axial T1W images of muscles of patient. **(A)** Arrows point to complete fatty atrophy in the gluteus maximus muscle bilaterally. **(B)** Arrows marks relative sparing of the right semitendinosus muscle. **(C)** Arrows mark hypertrophied short head of biceps femoris muscle bilaterally. **(D)** Arrows point to relatively hypertrophic soleus and gastrocnemius muscle bilaterally. **(E)** Normal muscles of the upper girdle.

## Discussion

In RYR1-related myopathy, intracellular calcium dysregulation and decreased protein expression leads to a heterogeneous presentation not only limited to malignant hyperthermia but also to a myopathy (9). Characteristic appearance features of KDS distinguish this disease from other neuromuscular disorders and should be a factor prompting diagnostic tests. Patients with King-Denborough syndrome show some typically myopathic traits (ptosis, high arched palate, elongated face), however, weakness in the muscles of the lower face is not typical for this group. Distinguishing features from other myopathies are low set ears and abnormal palpebral slant/ antimongoloid obliquity, found in more than half of analyzed patients. However, cryptorchidism seems to be the most important warning sign in KDS.

The similar set of dysmorphic features can be also found in Native American myopathy (NAM) autosomal recessive disorder also known as “congenital myopathy-cleft palate-malignant hyperthermia syndrome” or STAC3 disorder. (10) It should be emphasized that despite CK in KDS serum is frequently elevated (63%), in other RYR1 related myopathy could be normal. The most characteristic dysmorphic features also presented in our patient should lead the clinicians to correct diagnosis and genetic testing. Undiagnosed KDS disorder before qualification for anesthesia exposes the patient to a risk of malignant hyperthermia. This pharmacogenetic disorder is likely to be fatal if untreated. Mortality can be reduced from 80% to 1.4% when the specific drug is available—dantrolene.

In our study, we analyzed all described to date dysmorphic features characteristic for KDS and compared them with abnormalities found in our patient (Table 1). Our patient presents most of the clinical features described in other historically diagnosed KDS patients. Those facial features combined with skeletal abnormalities such as pectus excavatum are suggestive of KDS.

**Table 1.** Clinical features described in published KDS patients and our patients.

**Total number of analyzed cases:**	**15 (12 males / 3 females)**	**(Male)**

**Symptom name**	**Symptom occurrence**	**Case 1**
ptosis	12	+
lumbar lordosis	11	+
small build	10	+
cryptorchidism	10	+
antimongoloid obliquity	10	+
low set ears	10	+
kyphosis/ kyphoscoliosis	9	+
malignant hyperthermia	9	−
pectus carinatum/excavatum	8	+
hypoplastic mandible	8	−
motor development delay	8	+
webbed neck	6	−
high arched palate	5	+
joint hyperextensibility	5	+
palpebral slant	4	−
crowded lower teeth	2	−
mental development delay	2	−
scapular winging	2	+
clinodactyly	2	+
long face	2	−
hypertelorism	2	+
extra fontanelle	1	−
hip dislocation	1	+
strabismus	1	−
pes cavus	1	+
protuberant nose	1	+
long upper lip	1	+
prominent philtrum	1	−
scaphocephaly	1	−
rocker bottom foot	1	−
swallowing problem	1	−
deep set eyes	1	+

In our view, the present case is best interpreted not as a phenotype-exclusive diagnosis outside the broader RYR1 spectrum, but rather as an **RYR1-related congenital myopathy with a King-Denborough-like phenotype**. This interpretation is based on the observation that the proband presented not only with relatively nonspecific myopathic features, such as hypotonia, delayed motor development, mildly elevated CK, and selective muscle MRI abnormalities, but also with a broader **syndromic constellation of dysmorphic and skeletal findings**. In particular, the coexistence of **ptosis, downslanting palpebral fissures, low-set ears, small build/ short stature, cryptorchidism, lumbar hyperlordosis, kyphosis/kyphoscoliosis, and pectus deformity** suggests more than an isolated congenital myopathy and is more consistent with the classical clinical descriptions of King-Denborough syndrome, in which congenital myopathy is accompanied by characteristic craniofacial dysmorphism, orthopedic abnormalities, and malignant hyperthermia susceptibility. At the same time, we fully acknowledge that none of these findings is pathognomonic and that substantial phenotypic overlap exists both with the wider spectrum of RYR1-related myopathies, which may likewise combine congenital myopathy, ptosis, short stature, kyphoscoliosis, palatal abnormalities, and malignant hyperthermia susceptibility. For this reason, we cautiously opt to avoid overstatement and to frame the phenotype more cautiously as **KDS-like** rather than as an unequivocally distinct syndrome.

Importantly, once a pathogenic or likely pathogenic **RYR1** variant has been identified, the historical syndromic label becomes less important for clinical management than recognition of the underlying **RYR1-associated malignant hyperthermia risk**. Indeed, malignant hyperthermia susceptibility is well established not only in syndromic presentations such as King-Denborough syndrome, but also in **nonsyndromic** RYR1-related disease; therefore, the absence of a fully expressed or “classic” congenital myopathy phenotype does not eliminate anesthetic risk. Nevertheless, we deliberately chose to retain the discussion of King-Denborough syndrome because the phenotypic pattern observed in our patient may serve as a clinically useful warning sign, prompting consideration of malignant hyperthermia susceptibility and early **molecular testing of RYR1 in the proband as well as evaluation of at-risk relatives**. In this sense, recalling the KDS phenotype remains relevant not because it defines a rigid nosologic category, but because it may facilitate recognition of patients and families in whom perioperative risk would otherwise remain underappreciated.

The pattern observed in MRI scans including selective muscle involvement with relative sparing of the anterior tight compartment is highly typical for RYR-1 related myopathy. There are no specific MRI features for KDS.

As the MRI may reflect subclinical changes in case of our patient it precedes apparent muscle weakness or EMG changes. It underlines the importance of MRI in differential diagnosis in patients with RYR1 even in the early stages of the disease.

The recognition of a phenotype suggestive of malignant hyperthermia susceptibility has critical practical implications for this patient. The family was informed about the risk of MH (including the need for avoiding the use of halogenated violate anesthetics and succinylcholine) and referred for formal genetic counseling. According to current guidelines for RYR-1 related disorders, strict avoidance of potent volatile inhalational anesthetics and depolarizing muscle relaxants is required to prevent life-threatening MH crises. Future surgical procedures must be planned using safe alternatives, such as total intravenous anesthesia, combined with rigorous core temperature monitoring. Dantrolene should be available for planned procedures. (11,12,13,14)

The genetic background of KDS is not fully understood, the literature has presented cases conditioned by RYR1 mutations, both recessive and dominant (8).

## Conclusion

Considering the severity of the disorder and the high mortality risk due to a malignant hyperthermia, in differential diagnosis of children with myopathy and characteristic dysmorphic features King-Denborough syndrome should be considered. The RYR1 genetic testing should be performed at the beginning of diagnostic process and prior to the general anesthesia.

## Suppl 1.

List of 127 analyzed genes associated with dystrophies and elevated CK levels (ACADVL; ACTA1; AGL; AGRN; ALG14; ALG2; AMPD1; ANO5; B3GALNT2; BAG3; BICD2; BIN1; BVES; CACNA1S; CAPN3; CAV3; CHAT; CHKB; CHRNA1; CHRNB1; CHRND; CHRNE; CHRNG; CLN3; COL4A1; COL4A2; COL6A1; COL6A2; COL6A3; COLQ; CPT2; CRYAB; DAG1; DES; DMD; DMPK; DNAJB6; DNM2; DPAGT1; DPM3; DYSF; EMD; ENO3; ETFB; ETFDH; FHL1; FKRP; FKTN; FLNC; GAA; GBE1; GFPT1; GMPPB; GNE; GOLGA2; GYG1; GYS1; HNRNPDL; INPP5K; ISCU; ISPD; LAMA2; LAMB2; LAMP2; LARGE; LDB3; LDHA; LIMS2; LMNA; LPIN1; MEGF10; MLTK; MSTO1; MUSK; MYH2; MYH7; MYO18B; MYOT; PFKM; PGAM2; PGK1; PGM1; PHKA1; PHKB; PLEC; PNPLA2; POGLUT1; POMGNT1; POMGNT2; POMK; POMT1; POMT2; POPDC3; PREPL; PRKAG2; PTRF; PYGM; PYROXD1; RAPSN; RBCK1; RYR1; SCN4A; SEPN1; SGCA; SGCB; SGCD; SGCG; SLC22A5; SLC25A20; SMCHD1; SPEG; SPTBN4; SYNE1; SYNE2; SYT2; TCAP; TIA1; TMEM43; TNPO3; TOR1AIP1; TRAPPC11; TRIM32; TRIM54; TRIM63; TTN; VCP; VMA21.
